# Development and validation of a clinical prediction model for *Aspergillus fumigatus* sensitization in adults with asthma: a retrospective study

**DOI:** 10.3389/fmed.2025.1640399

**Published:** 2025-10-22

**Authors:** Feifei Liu, Qi Tian, Shanling Yu, Chunmi Niu, Shufeng Xu

**Affiliations:** ^1^Department of Pulmonary and Critical Care Medicine, The First Hospital of Qinhuangdao, Qinhuangdao, China; ^2^Hebei Provincial Key Laboratory for Research on Pathogenesis and Long-term Management of Lung Cancer and Chronic Airway Diseases, Qinhuangdao, China; ^3^Intensive Care Unit, The First Hospital of Qinhuangdao, Qinhuangdao, China

**Keywords:** *Aspergillus fumigatus*, sensitization, asthma, prediction model, total IgE

## Abstract

**Background:**

*Aspergillus fumigatus* sensitized asthma (AFSA) is associated with severe exacerbations and progressive lung damage; however, diagnosis remains challenging in resource-limited settings owing to limited access to *Aspergillus*-specific IgE (*A. f-*sIgE) testing. We aimed to develop a clinical prediction model using routinely available biomarkers for AFSA identification.

**Methods:**

This retrospective study enrolled 92 adult patients with asthma at The First Hospital of Qinhuangdao between 2023 and 2025. Participants were classified into AFSA and non-AFSA groups. Candidate predictors (demographics and hematological parameters) were analyzed using Least Absolute Shrinkage and Selection Operator (LASSO) regression, with subsequent multivariable logistic regression. Performance was validated via receiver operating characteristic (ROC) curves, calibration plots, and decision curve analysis (DCA).

**Results:**

Among 92 patients (mean age 56.5 ± 12.8 years; 60.9% female), 44.6% (*n* = 41) had AFSA. LASSO selected five predictors: sex, monocyte percentage, monocyte absolute count, lymphocyte percentage, and total IgE (TIgE). Final model retained male sex (Odds Ratio [OR] = 10.688; 95% Confidence Interval [CI]: 1.661–152.999) and TIgE (OR = 1.006; 95% CI: 1.003–1.011). The model achieved excellent discrimination: training cohort (Area Under the Curve [AUC] = 0.96, sensitivity = 0.93, specificity = 0.92); validation cohort (AUC = 0.88, sensitivity = 0.75, specificity = 1.00). Sex-specific TIgE cutoffs (527.5 IU/mL [males], 906.1 IU/mL [females]) yielded 79.2% accuracy.

**Conclusions:**

The developed prediction model using gender and TIgE provides a practical, accessible tool for AFSA screening, overcoming diagnostic barriers in settings lacking *A. f*-sIgE testing. However, this model remains exploratory and requires multicenter external validation before widespread clinical implementation.

## Introduction

Asthma is a chronic respiratory disease, affecting 1%–29% of population globally. It contributes significantly to disease burden due to recurrent exacerbations, progressive lung function decline, and increasing healthcare costs (1). Recent epidemiological data from China indicate that over 45 million adults aged ≥20 years are affected by asthma (2). Among all asthma phenotypes, allergic asthma represents the most clinically significant subtype due to its high prevalence (3). Filamentous fungi, particularly *Aspergillus fumigatus* (*A. f*), are well-recognized aeroallergens implicated in allergic asthma pathogenesis. Sensitization to *A. f* (*A. f*-sensitization) elevates the risk of severe asthma development. Approximately 20%–30% of patients with severe asthma demonstrate fungal allergen sensitization, predominantly to *A. f* (4). Severe asthma with fungal sensitization (SAFS) constitutes a distinct clinical phenotype, characterized by markedly impaired pulmonary function, heightened symptom severity (5), elevated mortality risk from acute exacerbations (6), refractoriness to conventional pharmacotherapy (7), and an increased likelihood of life-threatening attacks requiring intensive care (8). Allergic bronchopulmonary aspergillosis (ABPA), a complex pulmonary disorder driven by intense *A. f*-sensitization, is characterized by refractory clinical manifestations and bronchiectasis (9, 10). Notably, *A. f*-sensitization is also prevalent in mild-to-moderate asthmatics with near-normal lung function (11). A recent systematic review (73 studies; 23,003 asthmatics) reported *A. f*-sensitization prevalence ranging from 1.6 to 73%, with a pooled estimate of 25.1% (12). Given this high prevalence, differentiating *A. f*-sensitized asthma (AFSA) from non *A. f*-sensitized asthma (non-AFSA) and implementing universal *A. f*-sensitization routine in tertiary care settings are critical (13, 14).

Currently, elevated serum *Aspergillus fumigatus*-specific IgE (*A. f*-sIgE) levels exceeding the diagnostic cutoff value of 0.35 kUA/L represent the most sensitive test for detecting *A. f*-sensitization (13). While an immediate skin sensitivity reaction following skin prick test or intradermal antigen injection demonstrates the presence of fungus-specific IgE, skin testing presents several limitations, including operator-dependent variability in test quality, batch-to-batch antigen inconsistency, and a theoretical risk of anaphylaxis (13, 15). Consequently, skin tests demonstrate inferior diagnostic performance compared to *A. f*-sIgE, leading the Delphi Expert Consensus Group (DECG) to recommend *A. f*-sIgE as the preferred screening tool for *A. f*-sensitization due to its superior sensitivity (99%−100%) relative to *Aspergillus* skin testing (88%–94%) (14, 16, 17).

Although the fluorescent enzyme immunoassay (FEIA) using the Phadia platform is the goldstandard for detecting *A. f*-sIgE (13), its limited availability in primary healthcare settings across China frequently delays the diagnosis and management of AFSA patients. To address this clinical gap, we developed and validated a novel predictive model based on routinely accessible clinical biomarkers, offering primary care physicians a practical tool for early screening and personalized therapeutic interventions.

## Materials and methods

### Patients

This retrospective cohort study enrolled asthma patients who underwent *A. f*-sIgE testing at The First Hospital of Qinhuangdao (a 2,500-bed tertiary care center in Hebei province) between August 2023 and January 2025. The Institutional Review Board granted ethical approval with waiver of informed consent, in accordance with the Declaration of Helsinki’s provisions for retrospective studies. Patients were classified into two groups based on sensitization status: the AFSA group and non-AFSA group.

### Study protocol

This study strictly adhered to the TRIPOD-AI (Transparent Reporting of a Multivariable Prediction Model for Individual Prognosis or Diagnosis with Artificial Intelligence) guidelines—a contemporary reporting framework that enhances the transparent documentation of clinical prediction models incorporating both traditional regression and machine learning approaches (18).

### Definition of *Aspergillus fumigatus* sensitization

This study adopted the diagnostic criteria for *A. f*-sensitization as described in published literature (19, 20). The diagnosis of *A. f*-sensitization was established by: serum *A. f*-sIgE ≥ 0.35 kUA/L (ImmunoCAP^®^ Phadia 250 system, Thermo Fisher Scientific) (21, 22).

### Inclusion and exclusion criteria

**FIGURE 1:**
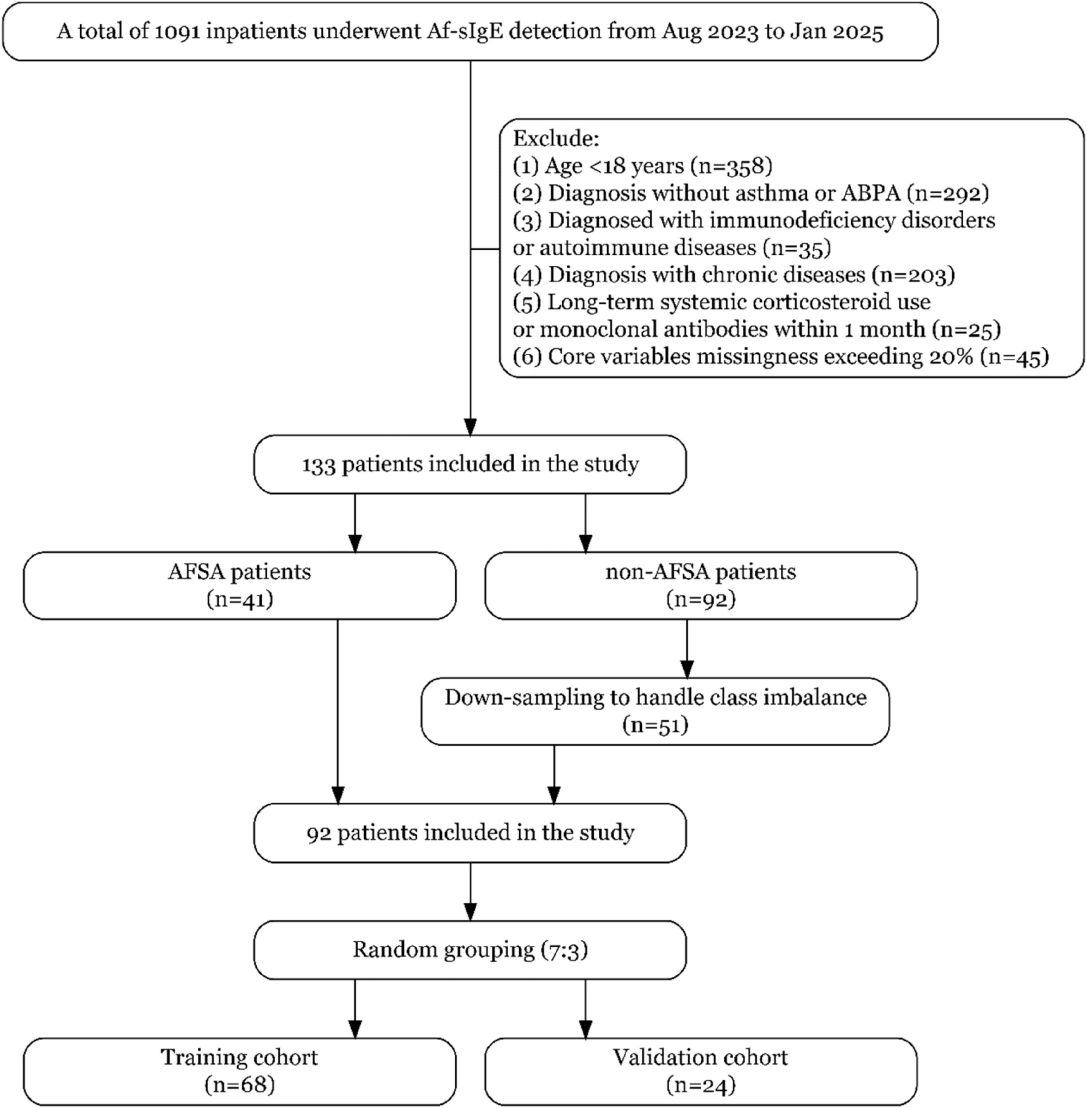
Flowchart of participant screening and enrollment.

Inclusion criteria: (1) Hospitalized patients at our tertiary referral center; (2) Age ≥18 years; (3) Diagnosis of asthma confirmed by the Global Initiative for Asthma (GINA) guidelines^1^; (4) At least one *A. f*-sIgE test performed during hospitalization. Exclusion criteria: (1) Primary or secondary immunodeficiency disorders or autoimmune diseases; (2) Comorbid chronic conditions, including tuberculosis, chronic obstructive pulmonary disease, coronary heart disease, hypertension, diabetes, or malignancies, as well as long-term systemic corticosteroid use (>5 mg prednisone-equivalent/day for ≥4 weeks) or monoclonal antibody therapy within the preceding 1 month; (3) Missing data exceeding 20% for core variables. Figure 1 demonstrates the participant screening flow, with quantitative documentation of exclusion criteria at each selection stage.

### Data collection and potential predictors

After reviewing relevant literature and applying clinical judgment, we collected the following data: demographic characteristics (gender and age) and hematological parameters including total IgE (TIgE), monocyte percentage (MONO%), monocyte absolute count (MONO#), lymphocyte percentage (LY%), and lymphocyte absolute count (LY#), basophil percentage (BASO%), and basophil absolute count (BASO#), eosinophil percentage (EOS%) and eosinophil absolute count (EOS#), neutrophil percentage (NEUT%) and neutrophil absolute count (NEUT#) from the 7-day period before and after the *A. f*-sIgE test. For repeated measurements, the highest recorded values were used for analysis. Following the exclusion of variables with >20% missing data, the multiple imputation method was employed to address remaining missing information. Specifically, for variables with a missing rate <20% (e.g., TIgE: 6.2% missing rate; MONO#: 7.1% missing rate), missing values were imputed using the “Multiple Imputation by Chained Equations (MICE)” method —implemented via the “mice” package in R^2^ —which generated 5 complete datasets based on standard MICE parameter settings. Serum samples were analyzed for TIgE and *A. f*-sIgE using the ImmunoCAP 1000 system (Thermo Fisher Scientific Inc.).

### Prediction model development

To mitigate potential biases associated with training on an imbalanced dataset (23), we used downsampling techniques to address class imbalance. Considering the positivity rate of *A.f*-sensitization, we utilized stratified sampling to allocate participants into training and validation cohorts (ratio 7:3). Subsequently, we applied LASSO regression to the training cohort to identify candidate predictors, which effectively mitigates multicollinearity by performing automated feature selection through coefficient shrinkage that reduces redundant variables to zero. Our analysis followed a two-stage approach: (1) LASSO-selected predictors were evaluated using multivariable logistic regression (Model 1), and (2) only significant predictors (*P* < 0.05) from Model 1 were retained in a refined multivariable model (Model 2). Finally, the optimal prediction model was determined via analysis of variance (ANOVA) and externally validated in the independent validation cohort.

### Performance of the prediction model

The prediction model’s performance was evaluated using the area under the receiver operating characteristic (ROC) curve (AUC), which was calculated with 95% confidence intervals (CI) via DeLong’s method. This metric quantifies the model’s discriminative ability to differentiate between AFSA and controls. Bootstrap-corrected calibration curves (500 resamples) were generated, and the Hosmer-Lemeshow goodness-of-fit test was applied to assess the agreement between predicted and observed probabilities. Additionally, decision curve analysis (DCA) was performed across threshold probabilities ranging from 0 to 100%, comparing the net benefit of the prediction model against “treat-all” and “treat-none” strategies to guide clinical decision-making.

### Statistical analysis

Continuous variables with a normal distribution were expressed as mean (standard deviation [SD]), while non-normally distributed (skewed) data were reported as median (interquartile range [IQR]). For intergroup comparisons, normally distributed variables were analyzed using the Student’s *t*-test, and non-normally distributed variables were assessed with the Mann–Whitney U test. Categorical variables were compared using the χ^2^ test or Fisher’s exact test when expected frequencies were <5. All statistical analyses were performed in R version 4.4.1 (R Foundation for Statistical Computing)^3^, utilizing the following packages: “glmnet” for LASSO regression, “pROC” for ROC analysis, “caret” for model training, and “rms” for nomogram development. A two-sided *P*-value < 0.05 was considered statistically significant.

## Results

### Demographic and clinical characteristics

Table 1 summarizes the demographic and clinical characteristics of the study cohort, which included 92 eligible asthma patients with a mean age of 56.5 (12.84) years (range: 18–87 years) and a sex distribution of 39.1% male and 60.9% female. Using stratified sampling based on sensitization status, participants were randomly allocated to either the training cohort (*n* = 68) or the validation cohort (*n* = 24). Among the total cohort, 44.6% (41/92) met the diagnostic criteria for AFSA, with baseline characteristics demonstrating homogeneity across the training and validation cohorts (Table 2).

**TABLE 1:** Baseline characteristics between the AFSA and non-AFSA groups.

Characteristic	AFSA *N* = 41^1^	Non-AFSA *N* = 51^1^	*p*-value^2^
Age	58 (15)	55 (11)	0.3
Sex			**0.003**
female	18 (44%)	38 (75%)	
male	23 (56%)	13 (25%)	
TIgE (0–60 kU/L)	1,673 (505, 3,908)	77 (25, 197)	**<0.001**
MONO% (3%–10%)	9.40 (7.30, 11.10)	9.30 (7.75, 11.05)	0.8
MONO# (0.1–0.6 × 10^9^/L)	0.82 (0.61, 1.02)	0.72 (0.56, 1.05)	>0.9
LY% (20%–50%)	30 (23, 42)	31 (28, 41)	0.2
LY# (1.1–3.2 × 10^9^/L)	2.12 (1.53, 3.38)	2.32 (1.84, 2.89)	0.6
BASO% (0%–1%)	0.90 (0.60, 1.50)	0.80 (0.50, 1.10)	0.2
BASO# (0–0.06 × 10^9^/L)	0.07 (0.05, 0.09)	0.06 (0.04, 0.08)	0.3
EOS% (0.4%–8.0%)	8 (4, 16)	7 (3, 12)	0.2
EOS# (0.02–0.52 × 10^9^/L)	0.55 (0.27, 0.86)	0.46 (0.24, 1.00)	0.7
NEUT% (40%–75%)	74 (59, 86)	78 (67, 88)	0.4
NEUT# (1.8–6.3 × 10^9^/L)	8.2 (3.7, 11.4)	7.5 (5.4, 11.5)	0.7

^1^Mean (SD); n (%); Median (IQR).

^2^Welch Two Sample *t*-test; Pearson’s Chi-squared test; Wilcoxon rank sum test. TIgE, total IgE; MONO%, monocyte percentage; MONO#, monocyte absolute count; LY%, lymphocyte percentage; LY#, lymphocyte absolute count; BASO%, basophil percentage; BASO#, basophil absolute count; EOS%, eosinophil percentage; EOS#, eosinophil absolute count; NEUT%, neutrophil percentage; NEUT#, neutrophil absolute count. Bold text indicates statistical significance (*p* < 0.05).

**TABLE 2:** Baseline characteristics of participants in training and validation cohorts.

Characteristic	Overall *N* = 92^1^	Training cohort *N* = 68^1^	Validation cohort *N* = 24^1^	*p*-value^2^
Age	57 (13)	57 (14)	56 (10)	0.8
Sex				0.8
female	56 (61%)	41 (60%)	15 (63%)	
male	36 (39%)	27 (40%)	9 (38%)	
TigE (0–60 kU/L)	241 (58, 1,137)	213 (39, 953)	407 (121, 1,445)	0.2
MONO% (3%–10%)	9.35 (7.45, 11.10)	9.40 (7.45, 11.10)	9.20 (7.58, 11.13)	0.8
MONO# (0.1–0.6 × 10^9^/L)	0.80 (0.57, 1.02)	0.80 (0.61, 1.01)	0.76 (0.51, 1.08)	0.6
LY% (20%–50%)	30 (24, 41)	31 (24, 42)	30 (24, 34)	0.5
LY# (1.1–3.2 × 10^9^/L)	2.26 (1.81, 3.04)	2.39 (1.77, 3.27)	2.21 (1.84, 2.64)	0.5
BASO% (0%–1%)	0.80 (0.50, 1.20)	0.80 (0.50, 1.20)	0.85 (0.50, 1.20)	0.6
BASO# (0–0.06 × 10^9^/L)	0.07 (0.05, 0.08)	0.07 (0.05, 0.08)	0.06 (0.05, 0.09)	0.9
EOS% (0.4%–8.0%)	8 (4, 15)	7 (3, 15)	8 (4, 14)	0.9
EOS# (0.02%–0.52 × 10^9^/L)	0.54 (0.24, 1.00)	0.54 (0.24, 1.01)	0.51 (0.19, 0.78)	0.5
NEUT% (40%–75%)	76 (64, 87)	79 (66, 86)	73 (60, 87)	0.6
NEUT# (1.8–6.3 × 10^9^/L)	7.8 (4.9, 11.5)	8.2 (5.3, 11.4)	7.5 (4.2, 11.8)	0.6

^1^Mean (SD); n (%); Median (IQR).

^2^Welch Two Sample *t*-test; Pearson’s Chi-squared test; Wilcoxon rank sum test. TIgE, total IgE; MONO%, monocyte percentage; MONO#, monocyte absolute count; LY%, lymphocyte percentage; LY#, lymphocyte absolute count; BASO%, basophil percentage; BASO#, basophil absolute count; EOS%, eosinophil percentage; EOS#, eosinophil absolute count; NEUT%, neutrophil percentage; NEUT#, neutrophil absolute count.

### Development of the prediction model

**FIGURE 2:**
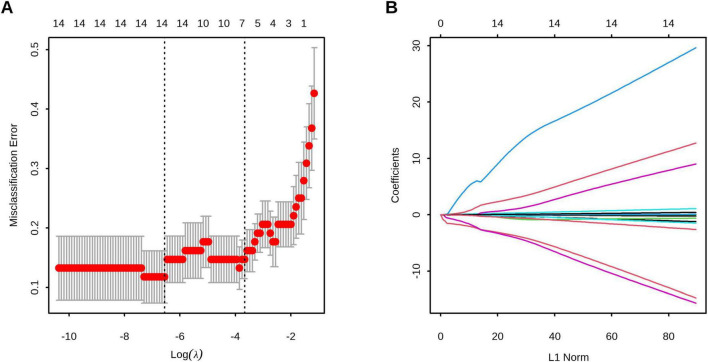
Feature selection using least absolute shrinkage and selection operator (LASSO) regression. **(A)** The optimal parameter (λ) selection in the LASSO model employed tenfold cross-validation using 1-standard error rule. The optimal values of λ are represented by dotted vertical lines. Among these values, λ = 0.026, corresponding to a logarithm of λ equal to –3.67, was selected as the optimal choice. **(B)** LASSO coefficient profiles of 13 clinical features. The optimal lambda value led to the identification of 5 features with non-zero coefficients.

We first performed preliminary screening using LASSO regression to identify potential predictors (Figures 2A, B), which yielded five candidate variables: Gender, MONO#, MONO%, LY%, and TIgE. These predictors were subsequently included in a multivariable logistic regression analysis (Model 1). Using the independent predictors identified in Model 1—male gender (OR = 15.688, 95% CI, 1.719–447.188) and TIgE (OR = 1.008, 95% CI, 1.004–1.016)—we constructed another multivariable logistic regression model (Model 2) and compared both models using ANOVA. The superior model (Model 2) was selected as the final prediction model and used to develop a nomogram, which incorporated two significant predictors: male gender (OR = 10.688, 95% CI, 1.661–152.999), and TIgE (OR = 1.006, 95% CI, 1.003–1.011) for predicting AFSA. The detailed multivariate analyses for the training cohort are presented in Table 3.

**TABLE 3:** The prediction model with multivariate logistic regression.

	Model 1			Model 2		
Characteristic	OR^1^	95% CI^1^	*p*-value	OR^1^	95% CI^1^	*p*-value
TIgE	1.01	1.00, 1.02	**0.007**	1.01	1.00, 1.01	**<0.001**
Sex (male)	15.7	1.72, 447	**0.038**	10.7	1.66, 153	**0.030**
LY%	0.90	0.80, 1.01	0.058			
MONO#	0.06	0.00, 2.05	0.2			
MONO%	1.20	0.89, 1.72	0.3			

^1^OR = Odds Ratio, CI = Confidence Interval. LY%, lymphocyte percentage; MONO%, monocyte percentage; MONO#, monocyte absolute count. Bold text indicates statistical significance (*p* < 0.05).

## Validation of the prediction model

### Discrimination

**FIGURE 3:**
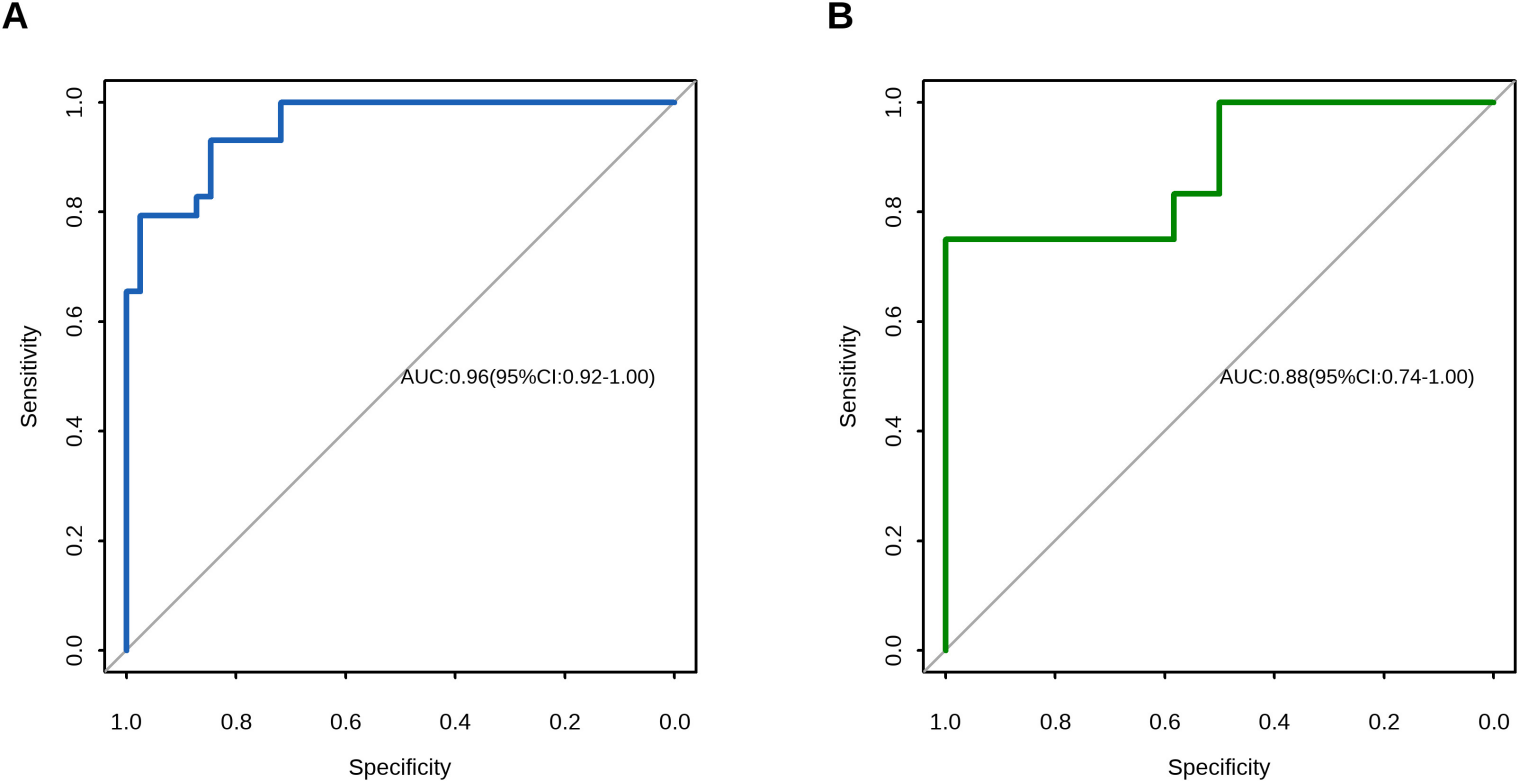
Receiver operating characteristic (ROC) curve analysis of the prediction model. **(A)** Training cohort demonstrated an area under the curve (AUC) of 0.96 (95% CI, 0.92–0.99). **(B)** Validation cohort showed an AUC of 0.88 (95% CI, 0.74–1.00). No significant difference was observed between the training and validation cohorts by DeLong’s test (*P* = 0.326), indicating preserved discriminative performance.

The model demonstrated robust discriminative performance, with AUC values of 0.96 (95% CI, 0.92–0.99) in the training cohort and 0.88 (95% CI, 0.74–1.00) in the validation cohort. Optimal cutoff probabilities were identified as 0.29 (95% CI, 0.16–0.69) and 0.78 (95% CI, 0.03–0.80) for the training and validation cohorts, respectively. DeLong’s test confirmed no significant difference in ROC curve performance between cohorts (*P* = 0.326). Sensitivity reached 0.93 (95% CI, 0.76–1.00) in the training cohort and 0.75 (95% CI, 0.50–1.00) in validation cohort, while specificity was 0.92 (95% CI, 0.72–1.00) and 1.00 (95% CI, 0.58–1.00), respectively (Figure 3 and Table 4).

**TABLE 4:** Performance of the model in training and validation cohorts.

Dataset	Cutoff	AUC	Sensitivity	Specificity	PPV	NPV	Accuracy
Training cohort	0.29 (0.16,0.69)	0.96 (0.92,1.00)	0.93 (0.76,1.00)	0.92 (0.72,1.00)	0.88 (0.72,1.00)	0.94 (0.84,1.00)	0.882
Validation cohort	0.78 (0.03,0.80)	0.88 (0.74,1.00)	0.75 (0.50,1.00)	1.00 (0.58,1.00)	1.00 (0.71,1.00)	0.80 (0.67,1.00)	0.792

PPV, positive predictive value; NPV, negative predictive value; AUC, area under the ROC curve.

### Calibration of the prediction model

**FIGURE 4:**
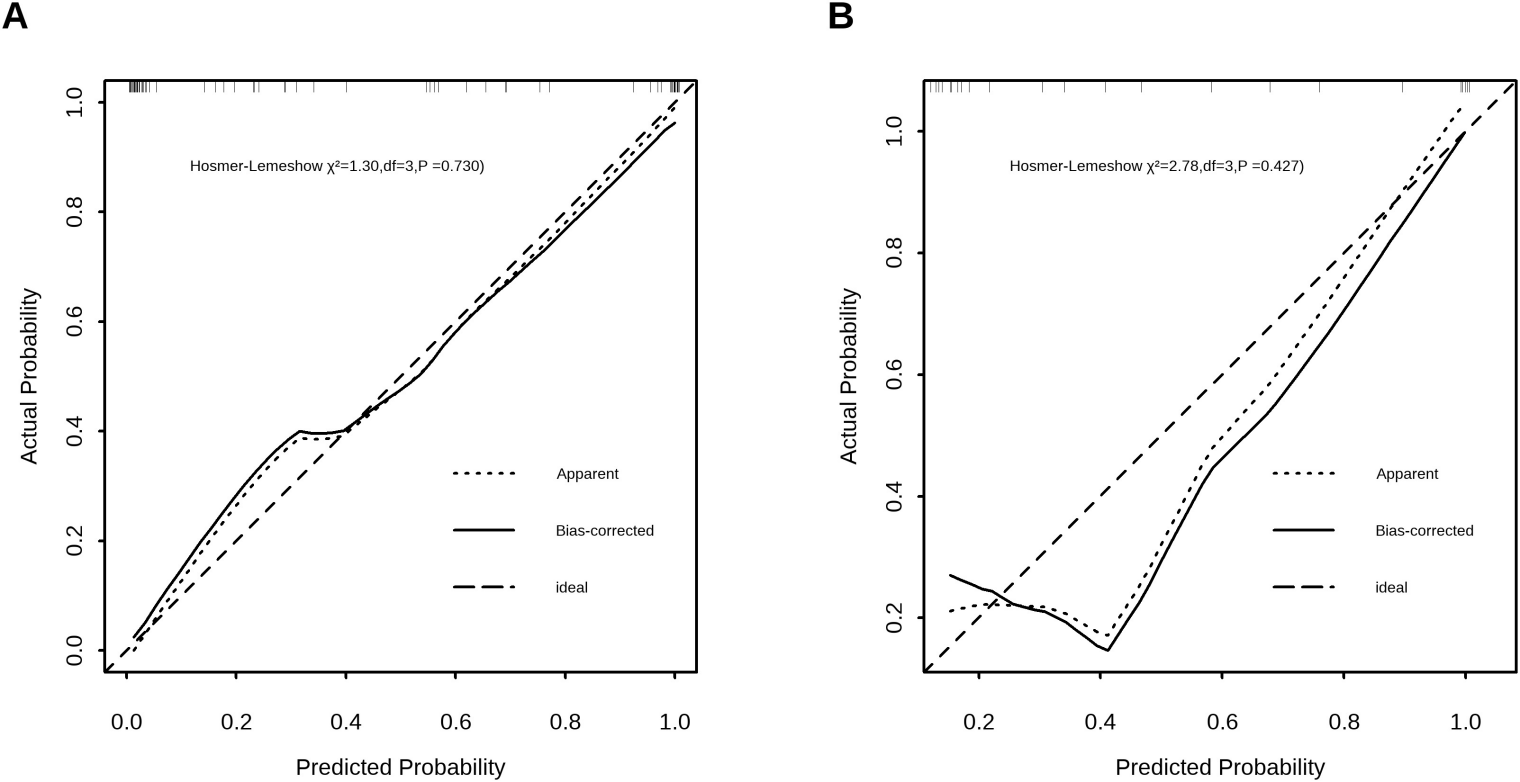
Calibration curves assessing the agreement between predicted probabilities and observed probabilities of *A.f*-sensitization in the training cohort **(A)** and the validation cohort **(B).** Hosmer-Lemeshow test revealed no significant divergence in both cohorts (Training: χ^2^ = 1.3, *P* = 0.730; Validation: χ^2^ = 2.78, *P* = 0.427).

The calibration plots showed excellent agreement between the predicted and observed probabilities of *A. f*-sensitization in both the training (Hosmer-Lemeshow χ^2^ = 1.3, df = 3, *P* = 0.73) and validation (Hosmer-Lemeshow χ^2^ = 2.78, df = 3, *P* = 0.427) cohorts, demonstrating robust model fit (Figure 4).

### Clinical use

**FIGURE 5:**
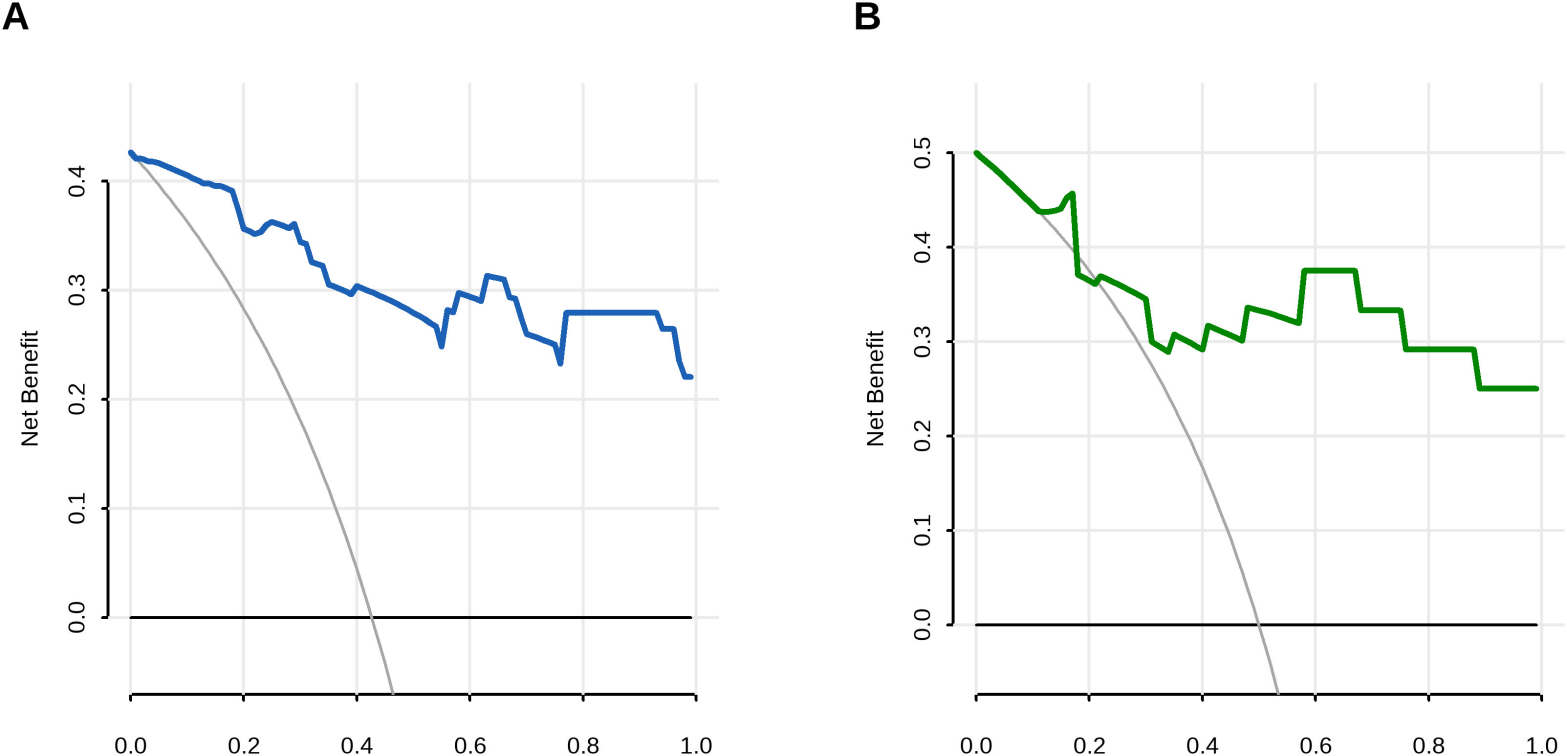
Decision curve analysis (DCA) evaluating the clinical utility of the prediction model. **(A)** Training cohort demonstrated superior net benefit across threshold probabilities 0.01–0.99 compared to extreme scenarios: all-negative strategy (black line) and all-positive strategy (gray line). **(B)** Validation cohort maintained clinical utility with superior net benefit at 0.12–0.99 risk thresholds.

**FIGURE 6:**
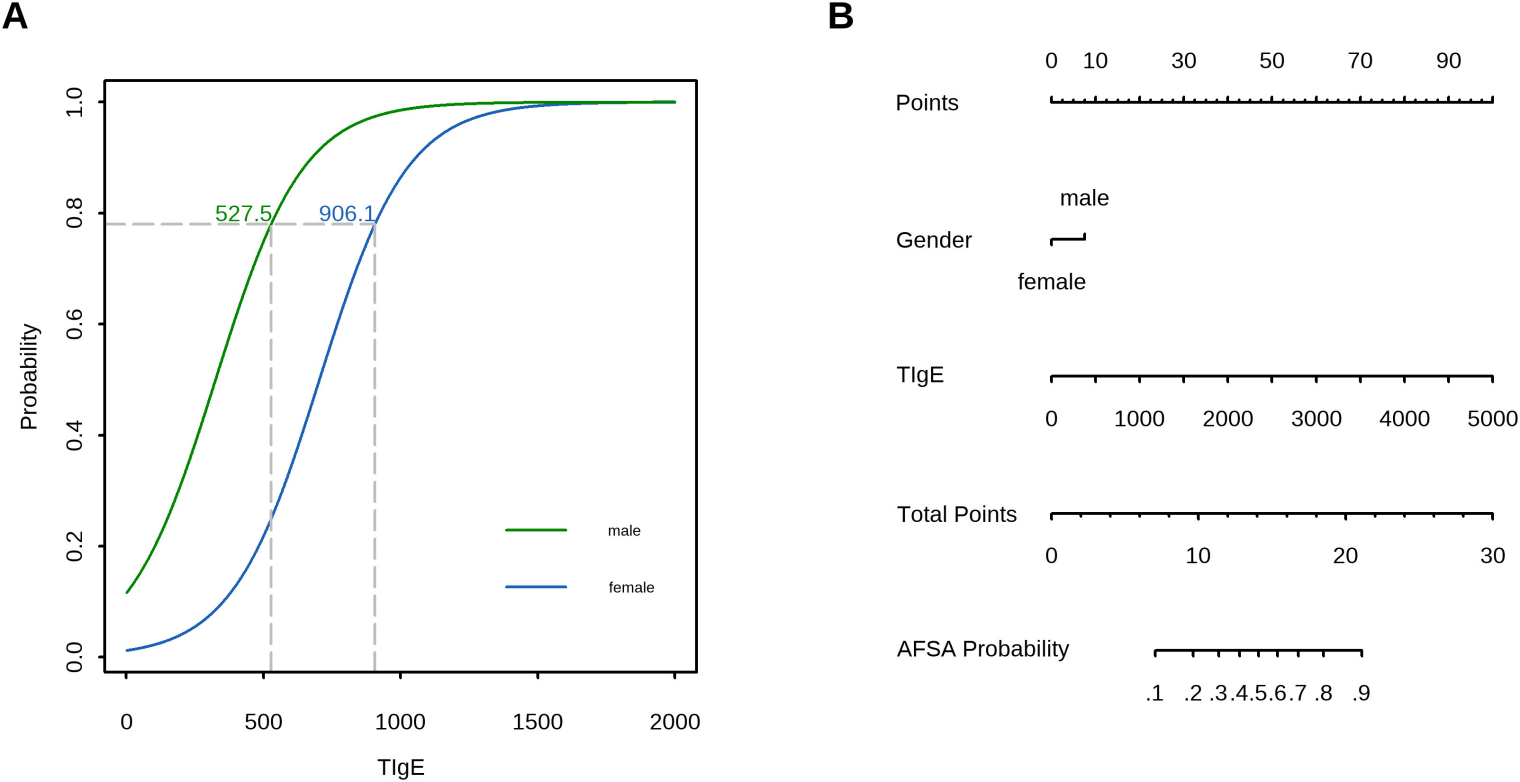
Clinical application of the prediction model: **(A)** In the validation cohort, TIgE using sex-specific thresholds (527.5 IU/mL for males and 906.1 IU/mL for females) demonstrated a sensitivity of 0.75 (95% CI, 0.50–1.00), specificity of 1.00 (95% CI, 0.58–1.00), and overall accuracy of 79.2%. **(B)** A nomogram for predicting AFSA based on the prediction model.

Decision curve analysis (DCA) confirmed the superior clinical utility of the model for predicting A. f sensitization, with significantly higher net benefit rates across threshold probabilities of 2%−99% in the training cohort and 12%−99% in the validation cohort compared to both “treat-all” and “treat-none” strategies (Figure 5). In the validation cohort, sex-specific TIgE thresholds (527.5 IU/mL for males and 906.1 IU/mL for females) demonstrated a sensitivity of 0.75 (95% CI, 0.50–1.00), specificity of 1.00 (95% CI, 0.58–1.00), and overall diagnostic accuracy of 79.2% for AFSA. The corresponding nomogram is presented in Figure 6.

## Discussion

We developed a novel diagnostic model based on conventional clinical biomarkers to accurately identify AFSA, thereby overcoming the limited availability of *A. f*-sIgE test in primary care settings. The model demonstrated high diagnostic accuracy and clinical practicality in differentiating AFSA from non-AFSA, thereby providing clinicians with an efficient and accessible tool to optimize therapeutic management and improve patient outcomes.

Our study exclusively included common laboratory parameters, a decision driven by its core objective: developing a clinically accessible predictive tool translatable to primary care and resource-limited settings. However, relying solely on laboratory indicators may omit more critical variables, whereas incorporating clinical and imaging variables could provide additional informative insights. For instance, demographic variables such as sex and region are associated with population genetic susceptibility, while factors like age and comorbidities directly relate to individual immune function—all key contributors to Aspergillus fumigatus-sensitized asthma (24–26). A predictive model for childhood allergic asthma integrating symptoms, age, and various IgE levels for airborne allergens achieved an AUC of 0.838 in the validation set (27). In contrast, another study showed that adding occupational exposure data improved risk stratification accuracy for A. flavus-sensitized conditions versus lab-only (IgE-based) models, as it identified “environmentally at-risk” individuals (e.g., workers in high-fungal-load settings) (28). Similarly, a model combining *A. f*-sIgE with HRCT-detected bronchiectasis had 22% higher specificity and 18% higher positive predictive value for ABPA severity stratification than lab-only models, directly guiding targeted interventions earlier models could not inform (29).

Nevertheless, non-laboratory variables pose challenges noted in prior studies. First, clinical variables (e.g., smoking history) have inconsistent documentation across centers, impairing model reproducibility. Second, HRCT has poor accessibility in primary care settings and highly subjective interpretation. These issues collectively limit the practical utility of non-laboratory indicators.

TIgE serves as a non-specific immunological marker with broad diagnostic applications, including helminth infections, HIV, and tuberculosis (30). In *A.f*-related pathologies, TIgE demonstrates particular clinical significance for diagnosing and monitoring ABPA (31), where consensus guidelines recommend ≥500 IU/mL as the diagnostic threshold and a ≥50% increase from baseline as an indicator of exacerbation—highlighting its dual role as both a diagnostic criterion and monitoring tool (14). Our findings further establish TIgE as a strong independent predictor of AFSA patients, consistent with its well-documented role in fungal-associated airway disease stratification (median levels: 375 [IQR: 521] IU/mL in non-AFSA, 1569 [IQR: 3000] IU/mL in AFSA, and 3068 [IQR: 4575] IU/mL in ABPA) (32). Although our study did not categorize ABPA separately due to sample limitation, the results underscore TIgE’s critical role in disease classification. Mechanistically, TIgE elevation likely represents sustained T helper 2 (Th2) immune activation, wherein *A.f* proteases induce epithelial alarmins to stimulate IL-4/IL-13 production, thereby promoting B-cell class switching to IgE synthesis. This hyperproduction perpetuates a self-amplifying cycle via FcεRI-bound IgE on mast cells and basophils, enabling rapid reactivation upon fungal re-exposure and exacerbating type 2 inflammation through histamine and leukotriene release (13). Clinically, TIgE’s widespread availability and high sensitivity render it valuable for initial *A. f*-sensitization screening, though its limited specificity—due to cross-reactivity with other allergens (e.g., pollens, helminths) and conditions like hyper-IgE syndrome—requires contextual interpretation (33).

In contrast to TIgE, which shows strong correlations with AFSA, our data revealed that peripheral blood eosinophils, neutrophils, basophils, lymphocytes, and monocytes displayed no significant differences between AFSA and non-AFSA groups, suggesting their minimal value in differentiating these clinical conditions. While AFSA is clearly a type 2 inflammatory disorder driven by eosinophilic responses (34), peripheral blood eosinophils possess poor sensitivity and specificity for detecting *A. f*-sensitization except in ABPA cases (35). Additionally, no meaningful correlation was found between eosinophil levels and either lung function parameters or other immunological markers (36). Rather than diagnostic utility, peripheral blood eosinophilia holds clinical relevance primarily for therapeutic decision-making, particularly regarding the use of anti-type 2 biological agents or combination therapy with prednisolone and itraconazole (37).

Monocytes, as innate immune cells that circulate in the bloodstream before migrating into tissues to differentiate into macrophages or dendritic cells, play crucial roles in host defense, inflammation, and immune regulation (38). Monocyte-derived macrophages may serve as key initial players in muco-obstructive lung diseases, particularly *A. f*-sensitized bronchiectasis and ABPA, where they act as primary detectors and defenders against *Aspergillus* (39). These cells detect fungal pathogens via pattern recognition receptors and initiate inflammatory responses through cytokine secretion, including tumor necrosis factor-alpha (TNF-α) and interleukin-1 beta (IL-1β), which subsequently recruit neutrophils and eosinophils in conditions like bronchiectasis and ABPA (40). In allergic asthma, bronchoalveolar lavage fluid monocyte counts increase following allergen exposure, with these cells interacting with T helper 2 (Th2) cytokines to potentially worsen mucus hypersecretion (41). Supporting this mechanism, a previous study reported significantly higher blood monocyte counts in AFSA patients with bronchiectasis (Mean: 439 [SD: 226] cells/μL) compared to non-sensitized controls (Mean: 329 [SD: 137] cells/μL) (32). While our LASSO regression analysis identified monocyte count and proportion as significant variables, these parameters failed to achieve statistical significance in logistic regression analysis, thereby excluding them from the prediction model. This discrepancy may reflect variations in disease severity among study populations.

Neutrophils exhibit a dual role in host defense against fungi and allergic airway inflammation, functioning as key effector cells that rapidly respond to infected lungs by suppressing *A.f* conidial germination through both phagocytosis and extracellular trap formation (42, 43), while simultaneously driving neutrophilic airway inflammation during acute asthma exacerbations. Experimental evidence shows that sensitized mice exposed to fungal conidia develop bronchial hyperreactivity accompanied by significant neutrophilic infiltration, underscoring the pivotal role of neutrophils at the nexus of antifungal immunity and allergic inflammation (42).

Basophils serve as pivotal effectors in allergic immune responses to *A.f*, bridging innate and adaptive immunity via their high-affinity IgE receptor (FcεRI) activation (44). Clinical studies reveal that basophils from *A.f*-sensitized patients display a hyper-responsive phenotype, demonstrating enhanced activation and degranulation upon allergen challenge relative to non-sensitized individuals (45). This functional priming is quantified by upregulated activation markers (e.g., CD203c, CD63), which are detectable via the basophil activation test (BAT) to accurately differentiate fungal colonization from true sensitization (46). Notably, the BAT has proven robust for ABPA diagnosis, exhibiting strong concordance with serologic markers (e.g., *A. f*-sIgE) and validated diagnostic criteria (45).

Lymphocytes play pivotal roles in type 2 inflammatory diseases, particularly allergic asthma and fungal-induced airway disorders. Type 2 innate lymphoid cells (ILC2s) drive eosinophilic asthma pathogenesis through IL-13 production, which compromises bronchial epithelial barrier integrity and induces airway hyperreactivity (47). In fungal infection contexts, adaptive Th2 lymphocytes orchestrate fibroblast recruitment and mediate tissue remodeling during allergic responses. Experimental models reveal time-dependent lymphocyte recruitment post-allergen challenge, peaking at day 7 alongside eosinophil surge (41). These observations underscore lymphocytes’ dual functionality—initiating early type 2 inflammation via ILC2s while propagating chronic Th2-driven fibrotic changes.

The observed absence of significant differences in peripheral blood leukocyte populations between AFSA and non-AFSA patients likely results from compartmentalized inflammation primarily localized to airways rather than systemic circulation, dynamic cellular recruitment patterns that standard clinical sampling cannot fully capture, or the dominant role of IgE-mediated pathways over cellular responses in AFSA pathogenesis (48, 49). Future research should investigate longitudinal cellular trafficking patterns, assess activation states using advanced techniques like flow cytometry for surface markers, perform compartment-specific analyses (e.g., bronchoalveolar lavage versus blood), and develop integrated biomarker panels incorporating cellular data with proteomic/transcriptomic profiles (50). Additionally, therapeutic targeting of specific cell subsets in well-characterized *A.f*-sensitized patients warrants exploration, given peripheral blood leukocyte counts demonstrate limited diagnostic value for AFSA in routine clinical practice.

Our study demonstrated a significantly higher proportion of males in the AFSA group compared with the non-AFSA group (56% vs. 25%, respectively), with multivariable logistic regression analysis confirming male sex as a independent predictive factor for *A. f*-sensitization (OR: 10.7). These findings are consistent with previous reports showing a male predominance in both AFSA and ABPA (73.8 and 71.4%, respectively) compared to non-sensitized asthmatics (54.8%, *P* = 0.012) (20, 51). In a recent retrospective analysis involving 2732 adult patients with asthma, sex male and black race were identified as high-risk factors for sensitization to various molds (52). This observed male predominance likely reflects the complex interaction of biological, environmental, and occupational factors.

Biologically, males inherently exhibit a subdued immune response compared to females, partly attributed to the lack of a second X chromosome—this genetic difference reduces the expression of immune-related genes, leading to weaker T cell activation and lower production of pro-inflammatory cytokines (e.g., IL-4, IL-13) that are critical for constraining fungal allergen-induced inflammation (53). Compounding this, testosterone, the primary male sex hormone, exerts distinct immunosuppressive effects: it attenuates B cell function and weakens the clearance of fungal spores, allowing persistent allergen exposure to trigger sensitization cascades (53). Environmentally, males are overrepresented in occupations (e.g., agriculture, construction) characterized by high *A. f* spore concentrations, resulting in more frequent and intense allergen contact than females (54–56). Unlike females who benefit from estrogen-mediated immune enhancement (supporting early fungal clearance), males lack such hormonal protection, making their immune systems less capable of counteracting repeated fungal exposure. Collectively, these factors—blunted baseline immunity, testosterone-driven suppression, and heightened environmental exposure—explain males’ greater risk of *A. f* sensitization, highlighting gender-specific mechanisms in fungal allergy pathophysiology.

Moreover, while sex appears to affect susceptibility, it does not appear to influence immunological markers of sensitization (e.g., total IgE levels, eosinophil counts, and Aspergillus-specific IgE/IgG) (57), highlighting the need for further research to better understand sex-specific risk factors while accounting for potential confounding variables such as healthcare access or regional allergen prevalence, and residual confounding needs to be further controlled through multivariable adjustment.

Notably, the CI for the OR of male sex is wide, which may be attributed to the small sample size of the study; therefore, the effect size of male sex on AFSA susceptibility should be interpreted with caution. It is anticipated that the CI of this OR will narrow significantly with an expanded sample size in future multicenter studies, which will enable more robust and reliable estimation of the effect size, thereby validating the association between male sex and AFSA risk.

In clinical practice, the prediction model and *A. f*-sIgE—the established gold-standard diagnostic test for AFSA—exhibit a complementary relationship rather than a substitutive one. Specifically, the model can be integrated into clinical practice across two key healthcare settings, with applications aligned to real-world resource constraints and diagnostic needs. In primary hospital settings—where access to *A. f*-sIgE testing remains frequently limited—it can be incorporated into routine assessment workflows for asthmatic patients. Male patients with TIgE levels exceeding 527.5 IU/mL and female patients with TIgE > 906.1 IU/mL should be classified as high-risk for AFSA. For these high-risk individuals, clinical recommendations include referral to tertiary hospitals for confirmatory *A. f*-sIgE testing; alternatively, empirical antifungal therapy may be considered in cases where standard asthma treatment fails to achieve adequate disease control. In tertiary hospital settings, the model functions as a pre-screening tool prior to *A. f*-sIgE testing, a role that helps reduce unnecessary diagnostic procedures, minimize healthcare expenditures, and streamline the diagnostic pathway for AFSA. Notably, the model functions as a screening tool rather than a diagnostic tool. For patients with elevated TIgE levels, clinicians should rule out other potential causes (e.g., recent parasitic infections, a history of pollen allergy) based on the patient’s detailed medical history. By enabling early identification of high-risk AFSA patients, the model facilitates timely initiation of targeted therapies, thereby reducing the frequency of acute asthma exacerbations and optimizing long-term patient outcomes.

## Limitations

This study has several limitations that require careful consideration. First, the model’s predictive accuracy may be compromised due to its inability to account for other sensitizing fungi or contributing factors, which could lead to a reduced positive predictive value and an increased risk of false-positive results. Second, the relatively small sample size and lack of multicenter validation limit the robustness and generalizability of the prediction model. Third, the absence of imaging features, such as high-resolution computed tomography (HRCT) findings, represents a notable gap, as these could enhance diagnostic accuracy by identifying structural abnormalities (e.g., bronchiectasis or mucus plugs) often associated with fungal sensitization (58). Additionally, the analytical approach focused exclusively on linear relationships, potentially missing non-linear interactions that could refine risk factor understanding and improve prediction. Notably, the non-specificity of TIgE may result in false-positive outcomes of the model; future studies could incorporate Aspergillus fumigatus-specific IgG (*A. f*-sIgG) or other parameters to improve the model’s specificity. Despite these constraints, the model remains clinically practical and sufficiently effective for its primary objective.

## Conclusion

In summary, our study fills a crucial clinical gap by developing a practical prediction model for AFSA using routine laboratory tests, thereby overcoming diagnostic limitations in resource-constrained settings where specialized fungal testing remains unavailable. While previous research has established the clinical significance of fungal sensitization in asthma severity and exacerbations, our model provides a translatable tool for primary care, enabling clinicians to assess *A. f*-sensitization risk during routine asthma evaluations without requiring additional infrastructure. This work paves the way for early identification of high-risk patients and targeted interventions in AFSA management. The clinical application of this model must be preceded by multicenter external validation; future studies should focus on multicenter validation across diverse populations and healthcare settings with a larger sample size and incorporate additional clinical and imaging indicators to further improve diagnostic accuracy.

## Data Availability

The raw data supporting the conclusions of this article will be made available by the authors, without undue reservation.

## Footnotes

1
http://ginasthma.org/

2
https://CRAN.R-project.org/package=mice

3
https://www.r-project.org/
